# Methyl 6-O-cinnamoyl-α-d-glucopyranoside Ameliorates Acute Liver Injury by Inhibiting Oxidative Stress Through the Activation of Nrf2 Signaling Pathway

**DOI:** 10.3389/fphar.2022.873938

**Published:** 2022-04-26

**Authors:** Qianqian Xu, Yanfang Deng, Jiaxiong Ming, Zengwei Luo, Xia Chen, Tianqi Chen, Yafen Wang, Shan Yan, Jiajun Zhou, Lina Mao, Weiguang Sun, Qun Zhou, Hong Ren, Yonghui Zhang

**Affiliations:** ^1^ Hubei Key Laboratory of Natural Medicinal Chemistry and Resource Evaluation, School of Pharmacy, Tongji Medical College, Huazhong University of Science and Technology, Wuhan, China; ^2^ Hubei Key Laboratory of Biotechnology of Chinese Traditional Medicine, National & Local Joint Engineering Research Center of High-throughput Drug Screening Technology, School of Life Sciences, Hubei University, Wuhan, China; ^3^ Biobank, Union Hospital, Tongji Medical College, Huazhong University of Science and Technology, Wuhan, China

**Keywords:** methyl 6-O-cinnamoyl-α-d-glucopyranoside, acute liver injury, oxidative stress, liver regeneration, hepatocyte apoptosis

## Abstract

Excessive stimulation of hepatotoxins and drugs often lead to acute liver injury, while treatment strategies for acute liver injury have been limited. Methyl 6-O-cinnamoyl-α-d-glucopyranoside (MCGP) is a structure modified compound from cinnamic acid, a key chemical found in plants with significant antioxidant, anti-inflammatory, and antidiabetic effects. In this study, we investigated the effects and underlying mechanisms of MCGP on acetaminophen (APAP)- or carbon tetrachloride (CCl_4_)-induced acute liver injury. As a result, MCGP inhibited cell death and apoptosis induced by APAP or CCl_4_, and suppressed the reactive oxygen species (ROS) generation stimulated by H_2_O_2_ in liver AML12 cells*. In vivo*, MCGP alleviated APAP/CCl_4_-induced hepatic necrosis and resumed abnormal aminotransferase activities and liver antioxidase activities. In addition, MCGP depressed APAP- or CCl_4_-induced oxidative stress through the suppression of CYP2E1 and activation of nuclear factor erythroid 2-related factor 2 (Nrf2) signaling pathway. MCGP also enhanced the number of PCNA-positive hepatocytes, increased hepatic PCNA and Bcl-XL, and decreased BAX expression in APAP-/CCl_4_-intoxicated mice. Furthermore, MCGP activated the GSDMD-N/cleaved caspase 1 pathway. In summary, MCGP might act as a potential therapeutic drug against drug-induced and chemical-induced acute liver injuries, and its underlying mechanisms might engage on the pressing of oxidative stress, refraining of hepatocyte apoptosis, and facilitating of liver regeneration.

## Introduction

The liver is necessary as a major site for metabolism, providing energy, and protein synthesis. In addition, the liver is a mediator of systemic and local innate immunity (Gu and Manautou 2012). Excessive stimulation of hepatotoxins and drugs often leads to acute liver injury, which may result in life-threatening clinical problems (Bernal and Wendon 2013). Acetaminophen (APAP) and carbon tetrachloride (CCl_4_) are two well-used hepatotoxins that can induce acute liver injury (Mossanen and Tacke 2015; Scholten et al., 2015). Acute liver injury induced by APAP overdose is reported to be the main cause for drug-induced acute liver injury (DILI) in many Western countries (Chun et al., 2009). APAP is a widely used antipyretic and analgesic drug, which can induce serious DILI when taken as overdose (Zheng et al., 2017). In addition, CCl_4_ induces oxidative damage, inflammation, fatty degeneration, and fibrosis in the liver. CCl_4_, an often-used solvent in organic chemistry, can also induce acute liver injury (Scholten et al., 2015). Nowadays, the acute liver injury models induced by these two compounds have been widely used to screen hepatoprotective agents.

Cytochrome P450 CYP2E1 is critical in oxidative stress, reactive oxygen species (ROS) generation, and hepatotoxic injury (Chowdhury et al., 2006; Zhai et al., 2008). APAP is metabolized mainly in the liver and transformed into a reactive metabolite N-acetyl-p-benzoquinone imine (NAPQI) by the liver CYP2E1 (Yan et al., 2018). In APAP overdose–induced liver injury, the overactivation of CYP2E1 depletes cellular glutathione (GSH), leading to oxidative stress and resultant liver damage (Lancaster et al., 2015). Similarly, CYP2E1 also catalyzes the generation of a free radical trichloromethyl (CCl_3_^−^) from CCl_4_ dehalogenation, thus inducing hepatotoxicity (Weber et al., 2003). CCl_3_^−^ reacts with oxygen to form trichloromethylperoxy (CCl_3_OO^−^) and stimulates oxidative stress resulting in calcium homeostasis and leading to apoptosis and necrosis (Weber et al., 2003).

Oxidative stress is also closely relevant with the nuclear factor erythroid 2-related factor 2 (Nrf2) signaling pathway in CCl_4_- or APAP-induced acute liver injury (Liu et al., 2013; Lu et al., 2016; Mitazaki et al., 2018). Nrf2 is a member of the Cap-n-collar basic leucine zipper family that regulates the expression of antioxidant genes, including superoxide dismutase (SOD), heme oxygenase 1 (HO-1), NAD(P)H quinine oxidoreductase 1 (NQO1), and glutathione S-transferase (GST) (Liu et al., 2013). The activation of the Nrf2 pathway protects liver injury from hepatotoxins such as APAP or CCl_4_ (Liu et al., 2013). Nrf2-deficient mice are highly susceptible to APAP hepatotoxicity (Enomoto et al., 2001). Thus, the Nrf2 signaling may be a promising target on suppression of oxidative stress for attenuating APAP- or CCl_4_-induced acute liver injury.

Natural products and their derivatives played a highly significant role in new drug discovery and development. In this concern, our group established compound libraries of natural products and their derivatives with nearly 6,000 compounds. Compounds with anticancer, antioxidative, hepatoprotective, anti-inflammatory, and antibacterial effects are focused in our screening work (Qiao et al., 2016; Xiang et al., 2016; Xu et al., 2021). Among these, we found an effective compound methyl 6-O-cinnamoyl-α-d-glucopyranoside (MCGP) with a pronounced hepatoprotective effect. MCGP is a glycosylation product of cinnamic acid, an organic acid occurring naturally in plants that have low toxicity and a broad spectrum of biological activities (Zhang et al., 2007). Cinnamic acid and its derivatives have been found to present potent anti-inflammatory and anticancer activities (Sova 2012; Ruwizhi and Aderibigbe 2020). In addition, MCGP also exhibited predominant hepatoprotective and antioxidative effects.

In this study, we determined the hepatoprotective effect of MCGP on APAP- or CCl_4_- induced liver injury through *in vivo* and *in vitro* experiments. Moreover, we uncovered the underlying mechanisms for the hepatoprotective effect of MCGP. Mechanistically, MCGP exerts a therapeutic effect through the pressing of oxidative stress, refraining of hepatocyte apoptosis, and facilitating of liver regeneration.

## Materials and Methods

### Materials

The general procedure for synthesis of MCGP (98% or higher purity) and its structure was characterized by one-dimensional nuclear magnetic resonance (NMR) spectrometer (Supplementary Materials). MCGP was dissolved in dimethyl sulfoxide (DMSO, Biosharp, China) to prepare a 40 mM stock solution and stored at −20°C before use for cell administration. MCGP was resuspended in 5% CMC-Na solution for the intragastric administration.

### Cell Culture, Administration, and Detection

Human liver cancer cell line 7,721 and HepG2, human hepatic stellate cell line LX-2, human monocytic leukemia cell line THP-1, and murine normal liver cell AML12 were purchased from Procell Life Science & Technology Co., Ltd. (Wuhan, China). Cells were cultured as monolayers in DMEM-high glucose (7,721 and HepG2)/DMEM (LX-2)/RIPM-1640 (THP-1) and D/F12 (AML12) medium (Hyclone, United States) supplemented with 10% fetal bovine serum (Procell, China) and 1% antibiotics of penicillin/streptomycin (100 units/mL) (Invitrogen, United States). Cells were grown in an atmosphere of 5% CO_2_ in air at 37 °C.

For the detection of the cytotoxicity effect, 25, 50, 100, 200, 400, 800, and 1,000 μM of MCGP were applied to 7,721, LX-2, and THP-1 cells. 25, 50, 100, 200, and 400 μM of MCGP were applied to HepG2 and AML12 cells for 48h. DMSO (0.1%) was used as control. Then, cell counting kit 8 (CCK8, Biosharp, China) was added to each well for 1–4 h, and absorbance was detected at 450 nm using a microplate reader (Thermo Fisher Scientific, United States).

For the cell viability assay, cells were incubated with 50, 100, and 200 μM of MCGP for 18 h followed by 30 mM of CCl_4_ for 6 h. Then, cell counting kit 8 was added to each well for 1–4 h, and absorbance was detected at 450 nm. For the cell apoptosis assay, cells were incubated with 50, 100, and 200 mM of MCGP for 18 h followed by 30 mM of CCl_4_ or 25 mM of APAP for 6 h. Then, the cells were determined using an Annexin V-fluorescein isothiocyanate (FITC) apoptosis detection kit (KeyGEN, Jiangsu, China) according to the manufacturer’s protocol. For the ROS generation assay, the cells were incubated with 50, 100, and 200 mM of MCGP administration for 18 h, after which 400 μM of H_2_O_2_ was introduced to generate ROS for 6 h. Then, the cells were fixed with 4% cold paraformaldehyde for 15 min. Thereafter, ROS (Sigma, United States) were stained and scanned using a digital microscopy scanner Pannoramic MIDI (3DHISTECH, Hungary).

### ABTS^•+^ Radical Inhibition Assay

To evaluate the antiradical activity of MCGP, 25–1,000 µM of MCGP was placed in 96-well plates (Corning, NY, United States), and 130 µl of 2,20-AzinoBis-(3-ethylbenzoThiazoline-6-Sulfonic acid) (ABTS^•+^) (Macklin, Shanghai, China) radicals was added. The ABTS^•+^ reagent was produced by reacting the ABTS solution (10 mM) with 30% H_2_O_2_ in advance. After incubation for 16 h at room temperature, 10 ml of this solution was diluted in 80 ml of sodium acetate hydrochloric acid buffer (Macklin, Shanghai, China). Ascorbic acid (25–1,000 µM) (Macklin, Shanghai, China) was used as a standard antioxidant. After 30 min of incubation in the dark at room temperature, the plates were read at 650 nm using a microplate reader (Thermo Fisher Scientific, United States).

### RNA-Seq and Data Analysis

Total RNAs were extracted with TRIzol reagent (Invitrogen, Thermo Fisher Scientific, United States) from HepG2 cells treated with 40 μM of MCGP or DMSO (control) for 24 h. Three biological replicates for the control group and the sample group were sequenced by Beijing Genomics Institute (BGI) using the BGISEQ-500 platform for each group. The differentially expressed genes (DEGs) were screened with a q value (adjusted *p* value) ≤ 0.01 and |fold change| ≥ 2. Bioinformatics Workflow including data filtering, mapping transcript prediction, differential gene expression analysis, Kyoto Encyclopedia of Genes and Genomes (KEGG) pathway analysis, and GO function analysis were performed by the platform established at BGI.

### Animal Administration

Specific-pathogen–free (SPF) male C57BL/6 and Balb/c mice (20–22 g) were purchased from Beijing HFK Bio-Technology Co. Ltd. (Beijing, China) and housed in a SPF environment at the experimental animal center of Tongji Medical College, Huazhong University of Science and Technology (Wuhan, China). After adapting feed for 1 week, mice were subjected to the following administration.

For the CCl_4_-induced acute liver injury, male C57BL/6 were randomly divided into 5 groups. 1) Control group (n = 10). 2) Model group (n = 10). 3) MCGP-M group (n = 10, 100 mg/kg). 4) MCGP-H group (n = 10, 200 mg/kg). 5) Positive group. (n = 10, 50 mg/kg of silymarin). Mice were first orally given MCGP once a day for 10 days, and then followed with a single intraperitoneal injection of 1% CCl_4_ (in olive oil, 5 ml/kg) 1 h after the last MCGP administration. Mice were killed 24 h after CCl_4_ injection, and plasma and liver tissue were collected.

For the acetaminophen (APAP)-induced acute liver injury, male Balb/c mice were randomly divided into 6 groups. 1) Control group (n = 10). 2) Model group (n = 10). 3) MCGP-L group (n = 10, 50 mg/kg). 4) MCGP-M group (n = 10, 100 mg/kg). 5) MCGP-H group (n = 10, 200 mg/kg). 6) Positive group (n = 10, 50 mg/kg of silymarin). Mice were first orally given MCGP once a day for 10 days, and then followed with intragastric administration of APAP (300 mg/kg). Mice were killed 6 h after APAP administration, and plasma and liver tissue were collected.

All mice were cared for and killed according to guidelines provided by the Institutional Animal Care and Use Committee of Tongji Medical College.

### Histology and Immunohistochemistry Analysis

The livers were fixed in 10% formaldehyde and embedded in paraffin. The liver tissue lesions were stained with hematoxylin and eosin (H&E) for histopathology. Immunohistochemistry was performed using CYP2E1 (Proteintech), BCL-xL (Abcam), PCNA, and Bax antibodies (Cell Signaling Technology). Aperio Image Scope software was applied to analyze the quantification of PCNA positive cells.

### Biochemical Assay

Whole-blood samples were allowed to coagulate for 15 min at room temperature and then centrifuged at 4,000 g at 4°C for 10 min to separate the serum. The liver homogenate was centrifuged at 12000 g, 4°C for 10 min. Serum concentrations of alanine aminotransferase (ALT), aspartate aminotransferase (AST), liver homogenate of glutathion (GSH), superoxide dismutase (SOD), and malondialdehyde (MDA) were detected with commercial test kits according to the manufacturer’s instructions (Nanjing Jiancheng Bioengineering Institute, Nanjing, Jiangsu, China).

### Quantitative Real Time PCR Tests

Total RNA was reverse-transcribed into cDNA using a transcription kit (ABP, United States). Quantitative RT-PCR (qRT-PCR) was performed using SYBR Green qPCR Mix (Vazyme Biotech Co., Ltd., China) with 0.2 μM forward and reverse primers in a final volume of 10 μl, and detected by ABI QuantStudio 5 (Thermo Fisher Scientific, United States). The resulting cDNA was amplified by incubating at 95°C for 5 min, 40 cycles of denaturation at 95°C for 10 s, annealing at 60°C for 20 s, and extension at 72°C for 30 s. Values were exhibited relative to β-actin. The corresponding primer sequences are listed in Supplementary Table S1.

### Western Blot Analysis

Total proteins from livers were lysed in radioimmunoprecipitation assay (RIPA, Beyotime, China) buffer, and 30 μg total proteins were used for each blot. The samples were separated by SDS-PAGE and transferred onto a nitrocellulose filter (NC, Millipore, United States) membrane by electroblotting. The membranes were blocked for 1 h in blocking buffer (Beyotime, China) and then incubated overnight with 1:1,000 dilutions of anti-Nrf2, anti-NQO1, anti-HO-1, anti-Keap1, anti-BCL-xL, anti-GSDMD, anti-caspase 1, anti-PCNA, anti-NLRP3, and anti-ASC (Cell Signaling Technology, United States) and 1:2,000 dilutions of anti-Bax, anti-IL1β, and anti-IL18 (Abcam, United States). After incubation with the secondary antibodies anti-mouse IgG (H + L) (DyLight™ 800, Cell Signaling Technology, United States) at 1:50,000 dilutions, membranes were imaged using a LiCor Odyssey scanner (LI-COR, United States). The protein expressions were normalized using β-actin as the reference (Cell Signaling Technology, United States) in the same sample.

### Terminal-Deoxynucleotidyl Transferase Mediated Nick End Labeling Assay

The liver tissues were fixed for at least 24 h in 4% paraformaldehyde, dehydrated in graded ethanol, and embedded in paraffin. The paraffin sections (5 μm) were incubated in a TUNEL reaction mixture from a kit according to the manufacturer’s instructions. The slides were scanned using a digital microscopy scanner Pannoramic MIDI (3DHISTECH, Hungary).

### Enzyme Linked Immunosorbent Assay

The liver homogenate was centrifuged at 12000 g, 4°C for 10 min. The liver homogenate was collected for IL1β and IL18 ELISA assay (Boster, Wuhan, China) according to the manufacturer’s instruction.

### Statistical Analysis

The experimental results are expressed as means ± SEM of at least triplicate measurements. The differences were evaluated by Student’s t-test with GraphPad Prism software (GraphPad Prism version 5.01 for Windows, Sand Diego, CA). The differences with *P and #*p* < 0.05 were considered statistically significant.

## Results

### Methyl 6-O-Cinnamoyl-α-d-Glucopyranoside Protected AML12 Cells From Acetaminophen or Carbon Tetrachloride–Induced Hepatotoxicity

**FIGURE 1:**
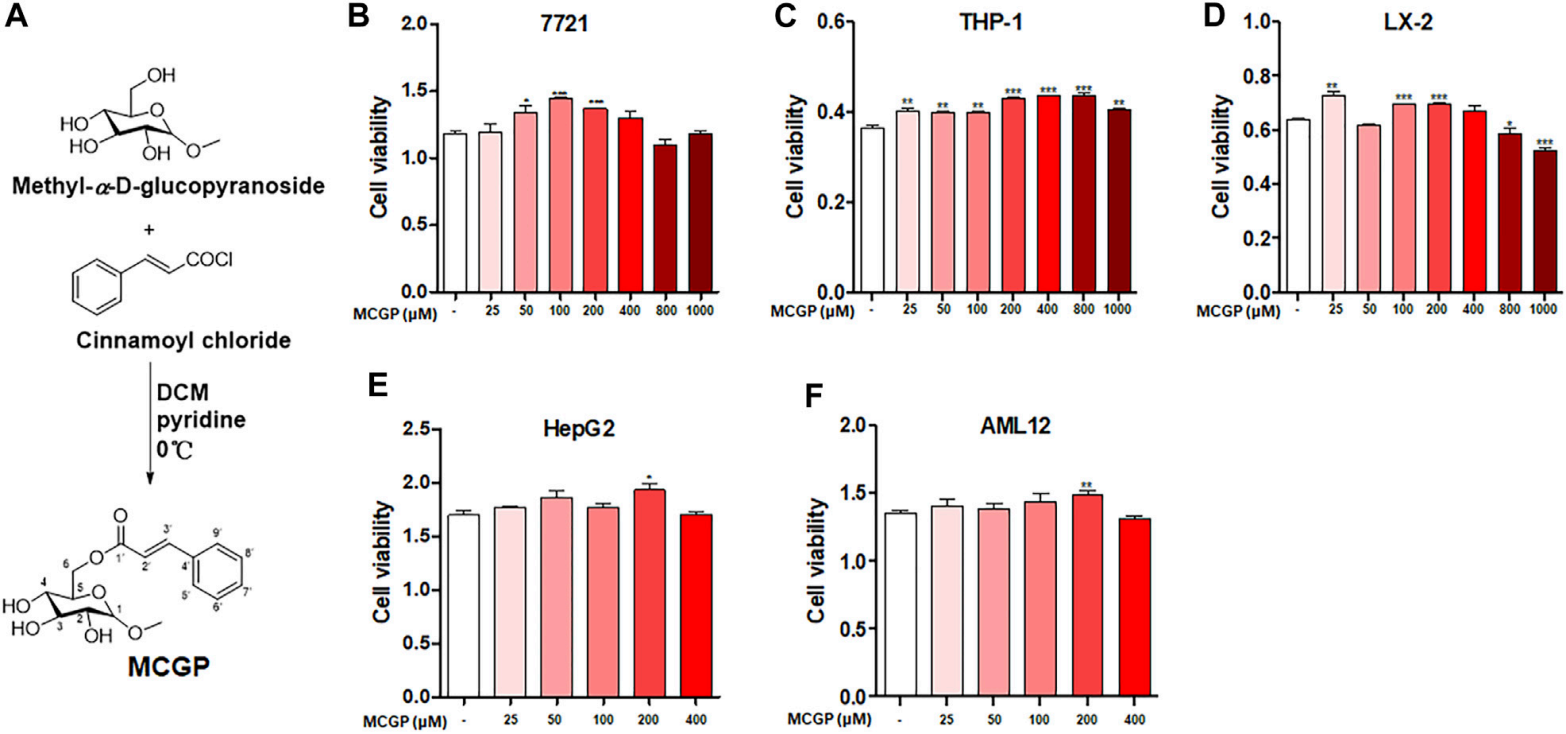
**(A)** Synthetic route and structure of MCGP. **(B-D)** Cytotoxicity of MCGP on 7,721 **(B)**, THP-1 **(C)**, and LX-2 **(D)** cells at 25–1,000 μM. **(E,F)** Cytotoxicity of MCGP on HepG2 **(E)** and AML12 **(F)** cells at 25–400 μM. Data were presented as the mean ± SEM. *p < 0.05, **p < 0.01, ***p < 0.001 vs control.

MCGP was synthesized according to the methods in Figure 1A. We first detected the cytotoxicity effect of MCGP on different cell lines. As a result, MCGP exhibited little cytotoxicity on 7,721 and LX-2 at dosages of 800 and 1,000 μM (Figures 1B,D). Interestingly, MCGP slightly promoted the cell growth at dosages of 25–400 μM at all the detected cell lines (Figures 1B–F). Accordingly, we presumed that MCGP might also promote cell growth in other conditions.

**FIGURE 2:**
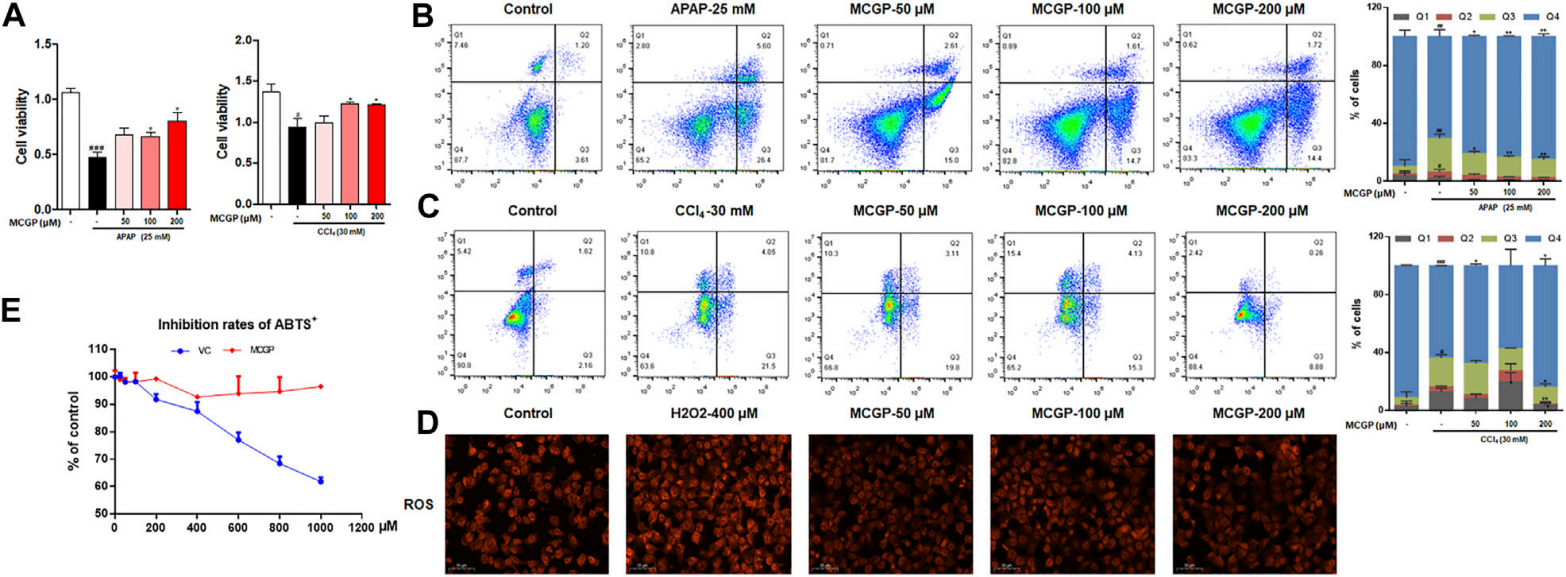
MCGP protected AML12 cells from APAP- or CCl_4_-induced hepatotoxicity. **(A)** The cell viability of AML12 cells co-treated with MCGP at 50–200 μM and 25 mM of APAP or 30 mM of CCl_4_. **(B,C)** Cell apoptosis of AML12 cells co-treated with MCGP at 50–200 μM and 25 mM of APAP **(B)** or 30 mM of CCl_4_ **(C)**. **(D)** The ROS generation of MCGP at 50–200 μM on AML12 cells stimulated by 400 μM of H_2_O_2_. **(E)** The inhibition rates of ABTS^•+^ of MCGP and ascorbic acid at 25–1,000 μM. Data were presented as the mean ± SEM. #*p* < 0.05, ##*p* < 0.01, ###*p* < 0.001 vs control, *p < 0.05, **p < 0.01 vs APAP or CCl_4_.

APAP and CCl_4_ are two well-used hepatotoxins that can induce hepatocyte apoptosis and liver injury *in vitro* and *in vivo*. Therefore, we identified the cell viability of AML12 cells co-treated by MCGP and APAP or CCl_4_. As expected, MCGP promoted the cell viability of AML12 cells treated by APAP or CCl_4_ (Figures 2A,B). Moreover, MCGP also inhibited cell apoptosis of AML12 cells injured by APAP or CCl_4_ (Figures 2C,D). In addition, we also found that MCGP could suppress the ROS generation induced by H_2_O_2_ (Figure 2E). This result prompted that MCGP might suppress oxidative stress by scavenging free radicals. However, MCGP exerted little effect on ABTS^•+^ radical inhibition (Figure 2F). Considering this, we supposed that MCGP could not directly scavenge free radicals, while it could scavenge through intracellular processes.

### RNA-Seq Reveals the Differentially Expressed Genes of Methyl 6-O-Cinnamoyl-α-d-Glucopyranoside–Administrated HepG2 Cells

**FIGURE 3:**
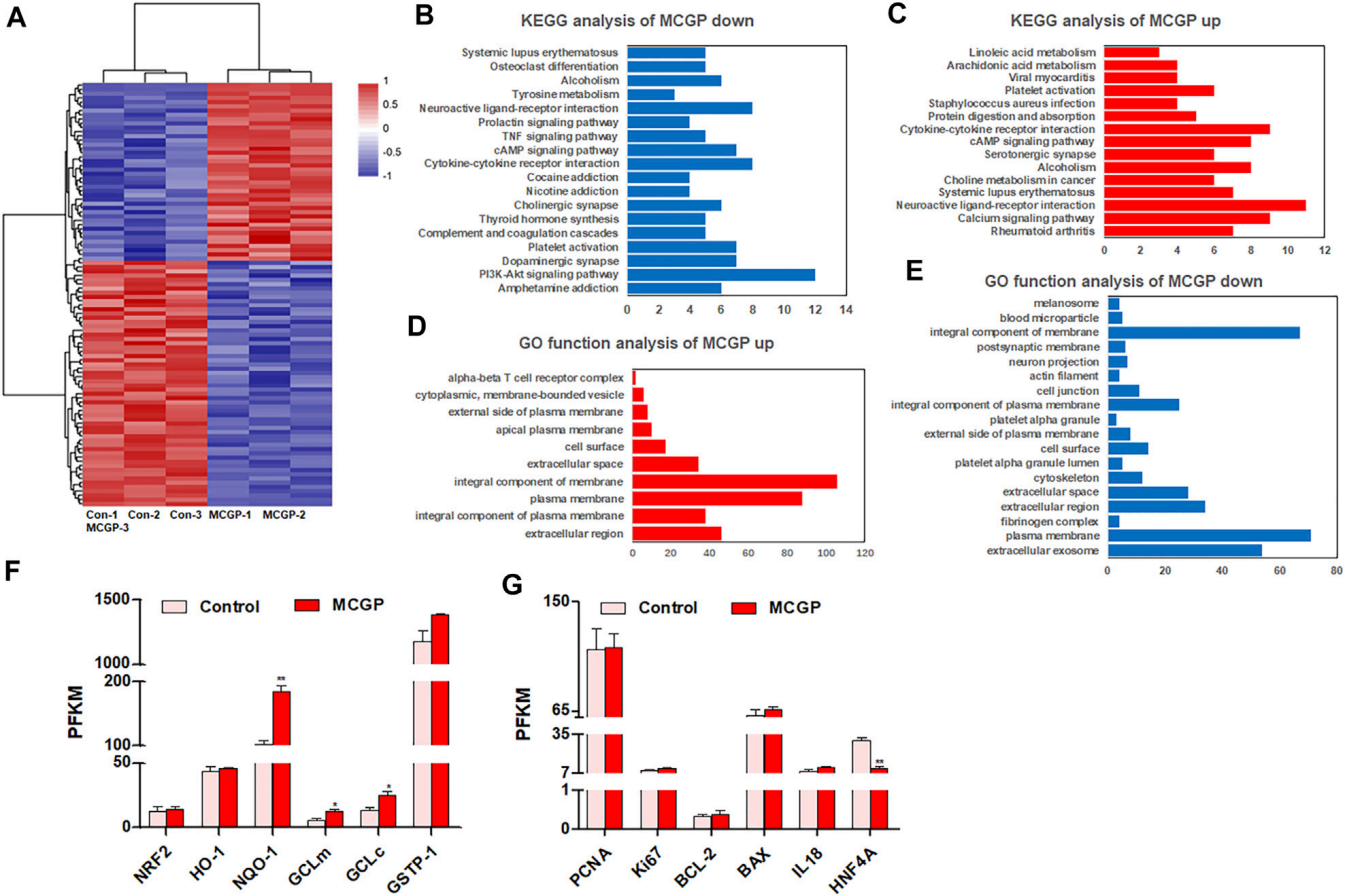
RNA-seq of control or MCGP-treated HepG2 cells. **(A)** The expression heat map of differentially expressed genes in MCGP-treated HepG2 cells. **(B)** KEGG pathway enrichment of MCGP upregulated genes. **(C)** KEGG pathway enrichment of MCGP downregulated genes. **(D)** GO function analysis of MCGP upregulated genes. **(E)** GO function analysis of MCGP downregulated genes. **(F,G)** The PKFM value of NFR2 signaling pathway components **(F)** and liver regeneration related genes of MCGP administration in HepG2 cells. *p < 0.05, **p < 0.01 vs control.

In order to comprehensively understand the effect of MCGP on liver cells, RNA-seq was conducted to compare the gene expression between 40 μM MCGP-treated HepG2 cells and DMSO control. As a result, 100 differentially expressed genes were screened between MCGP and control, including 58 down-expressed and 42 up-expressed genes regulated by MCGP (Figure 3A). The KEGG pathway analysis of these genes indicated an enrichment in metabolic pathways and PI3K-Akt signaling pathway (Figures 3B,C). The gene ontology (GO)-function analysis showed an enrichment in components of membranes (Figures 3D,E).

Oxidative stress, hepatocyte apoptosis, and liver regeneration are common processes engaged in APAP- or CCl_4_-induced hepatotoxicity (Scholten et al., 2015; Ramachandran and Jaeschke 2019). Hence, we screened the gene expression of related genes in RNA-seq results. As shown in Figure 3F, the Nrf2/HO-1/NQO1 signaling pathway components were almost upregulated by MCGP. In addition, MCGP also increased the liver regeneration–related genes PCNA, Ki67, BCL-2, and IL18, while it decreased hepatocyte nuclear factor 4 alpha (HNF4A) (Figure 3G). Taking together, we supposed that MCGP might protect the liver cell from APAP- or CCl_4_-induced hepatotoxicity through the activation of the Nrf2 signaling pathway. However, the effect and mechanism of MCGP *in vivo* still remains unknown.

### Methyl 6-O-Cinnamoyl-α-d-Glucopyranoside Treatment Alleviated Liver Injury and Oxidative Stress in Acetaminophen-/Carbon Tetrachloride-Intoxicated Mice

**FIGURE 4:**
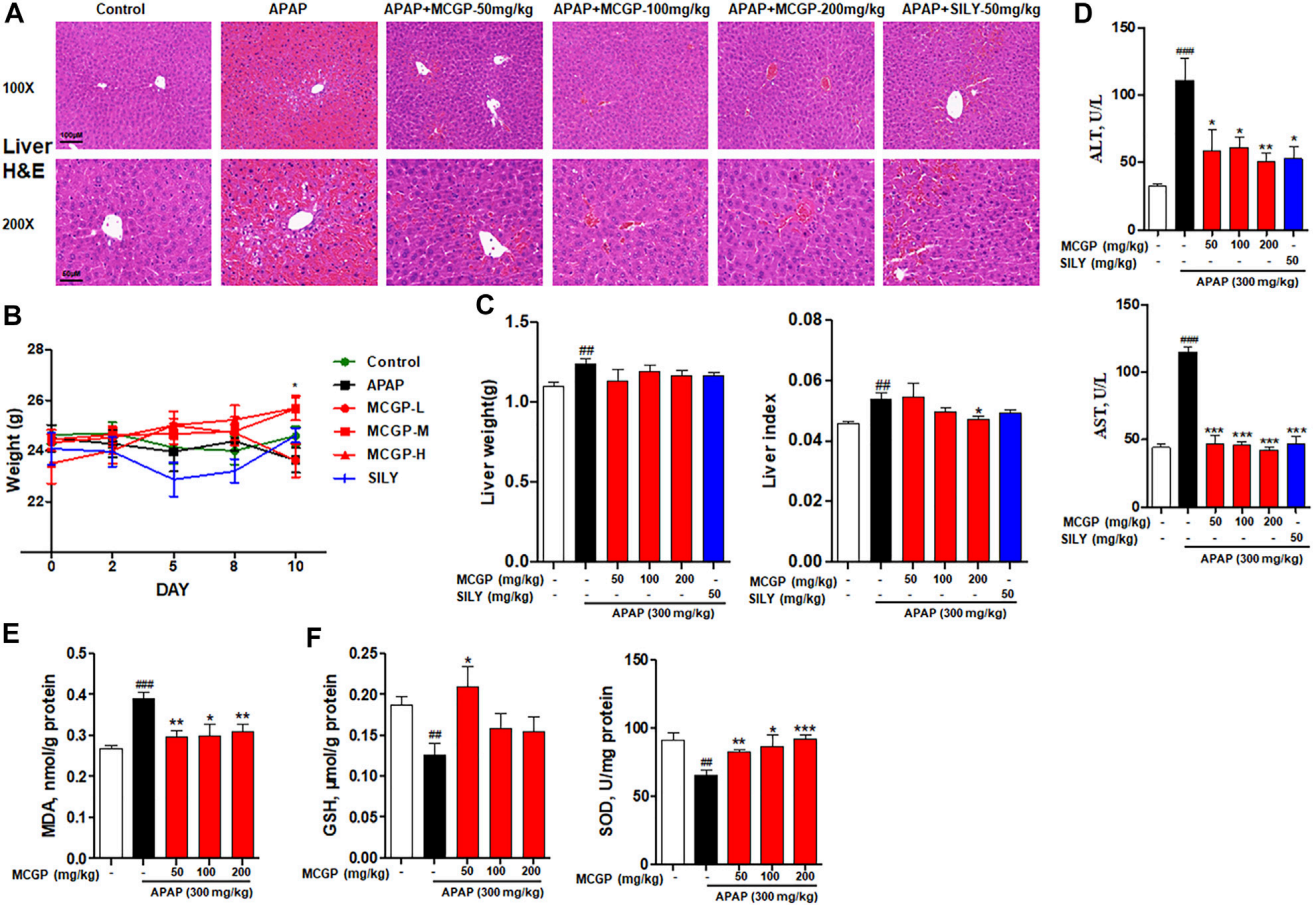
MCGP treatment alleviated APAP-intoxicated liver injury. **(A)** Liver histological observation. Mice were pretreated with vehicle or various dosages of MCGP (50, 100 or 200 mg/kg) or positive control silymarin once a day for 10 days, and then followed with intragastric administration of APAP (300 mg/kg). Typical images are chosen from each experimental group: Control, APAP, MCGP-L + APAP, MCGP-M + APAP, MCGP-H + APAP, SILY (silymarin + APAP). **(B)** Mice weight of each group. **(C)** Liver weight and liver index of each group. **(D)** Serum ALT and AST activity. **(E,F)** Liver levels of GSH, MDA, and SOD in each group. Data were presented as the mean ± SEM. ##*p* < 0.01, ###*p* < 0.001 vs control, *p < 0.05, **p < 0.01, ***p < 0.001 vs APAP.

**FIGURE 5:**
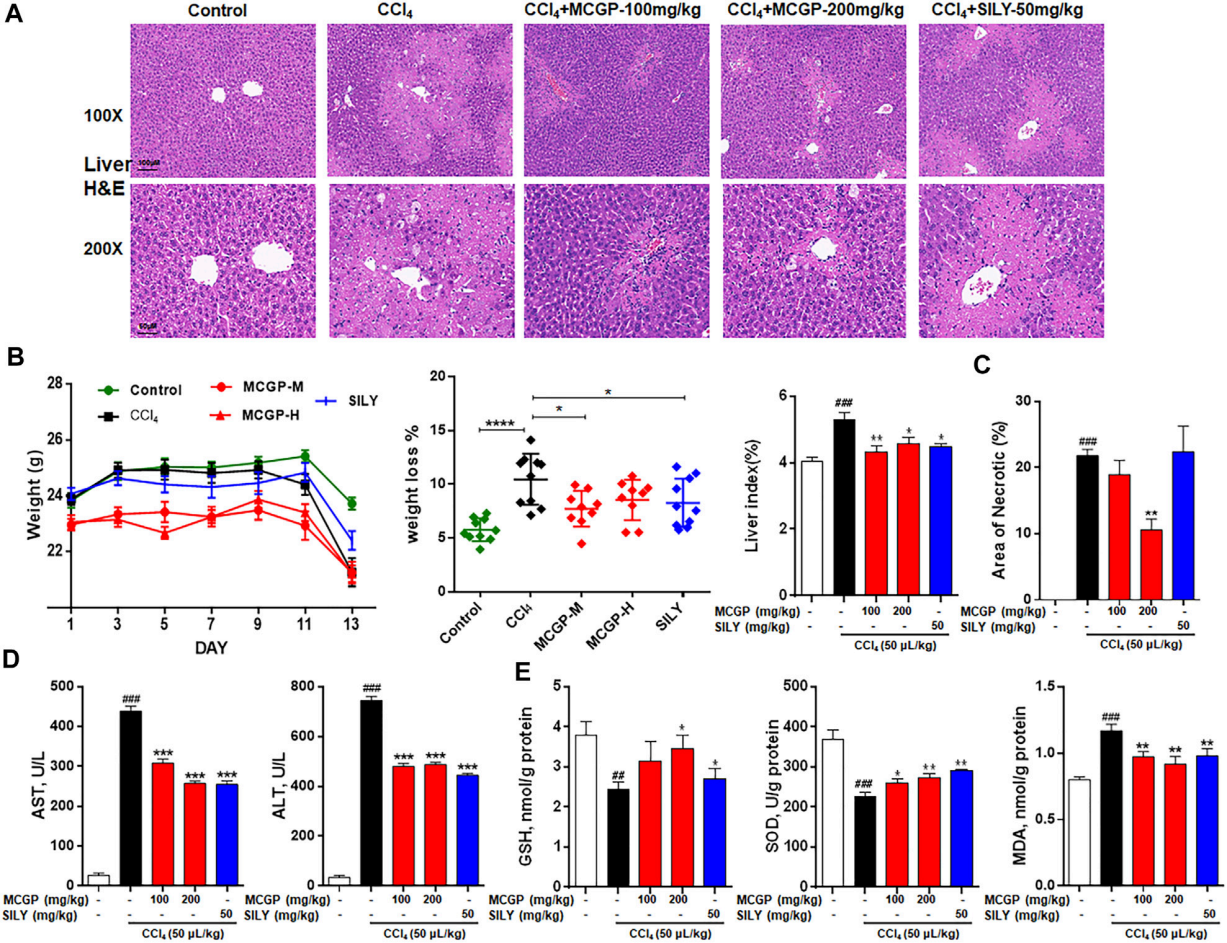
MCGP treatment alleviated CCl_4_-intoxicated liver injury. **(A)** Liver histological observation. Mice were pretreated with vehicle or various dosages of MCGP (100 or 200 mg/kg) or positive control silymarin (50 mg/kg) once a day for 10 days, and then followed with a single intraperitoneal injection of 1% CCl_4_ (in olive oil, 5 ml/kg) 1 h after the last MCGP administration. Typical images are chosen from each experimental group: Control, CCl_4_, MCGP-M + CCl_4_, MCGP-H + CCl_4_, SILY (silymarin + CCl_4_) (n = 10). **(B)** Mice weight, weight loss, and liver index of each group. **(C)** Counted necrotic areas of each group (n = 3). **(D)** Serum ALT and AST activity. **(E)** Liver levels of GSH, MDA and SOD in each group. Data were presented as the mean ± SEM. ##*p* < 0.01, ###*p* < 0.001 vs control, *p < 0.05, **p < 0.01, ***p < 0.001 vs CCl_4_.

To further verify the hepatoprotective effect of MCGP *in vivo*, we used APAP- or CCl_4_-induced liver injury mice models. As shown in Figure 4A, the liver histological evaluation in mice 6 h after APAP administration showed that pretreatment of MCGP alleviated APAP-induced intrahepatic hemorrhage and nuclear pyknosis in mice. Similarly, pretreatment of MCGP also improved liver damage in mice 24 h after CCl_4_ induction (Figure 5A). MCGP also inhibited the APAP-/CCl_4_-induced mice weight loss, and decreased liver weight and liver index (Figures 4B,C and Figure 5B). The liver necrotic areas were increased in CCl_4_-administrated mice, while MCGP reduced the increased necrotic areas (Figure 5C). MCGP also significantly inhibited the increase of the serum ALT/AST level in APAP-/CCl_4_-induced mice (Figure 4D and Figure 5D).

GSH, SOD, and MDA are critical markers indicating liver damage during APAP-CCl_4_-injured liver. GSH and SOD can protect cells from oxidative damage. Previous studies have revealed significant decrease in liver antioxidase activity GSH and SOD in APAP-/CCl_4_-induced acute liver injury (Xie et al., 2016). In addition, the lipid peroxidation product MDA was detected to reflect the liver oxidative damage (Ingawale et al., 2014). As a result, the activity of GSH and SOD were notably decreased in APAP-/CCl_4_-treated mice, accompanied by a significant increase in the MDA level. The pretreatment of MCGP improved GSH and SOD levels and inhibited the MDA level in both APAP- or CCl_4_-treated mice (Figures 4E,F and Figure 5E). Silymarin, a well-used hepatoprotective herbal drug, was used as the positive control.

### Methyl 6-O-Cinnamoyl-α-d-Glucopyranoside Treatment Inhibited CYP2E1 and Activated the Nrf2/HO-1 Signaling Pathway in Acetaminophen-/Carbon Tetrachloride-Intoxicated Mice

**FIGURE 6:**
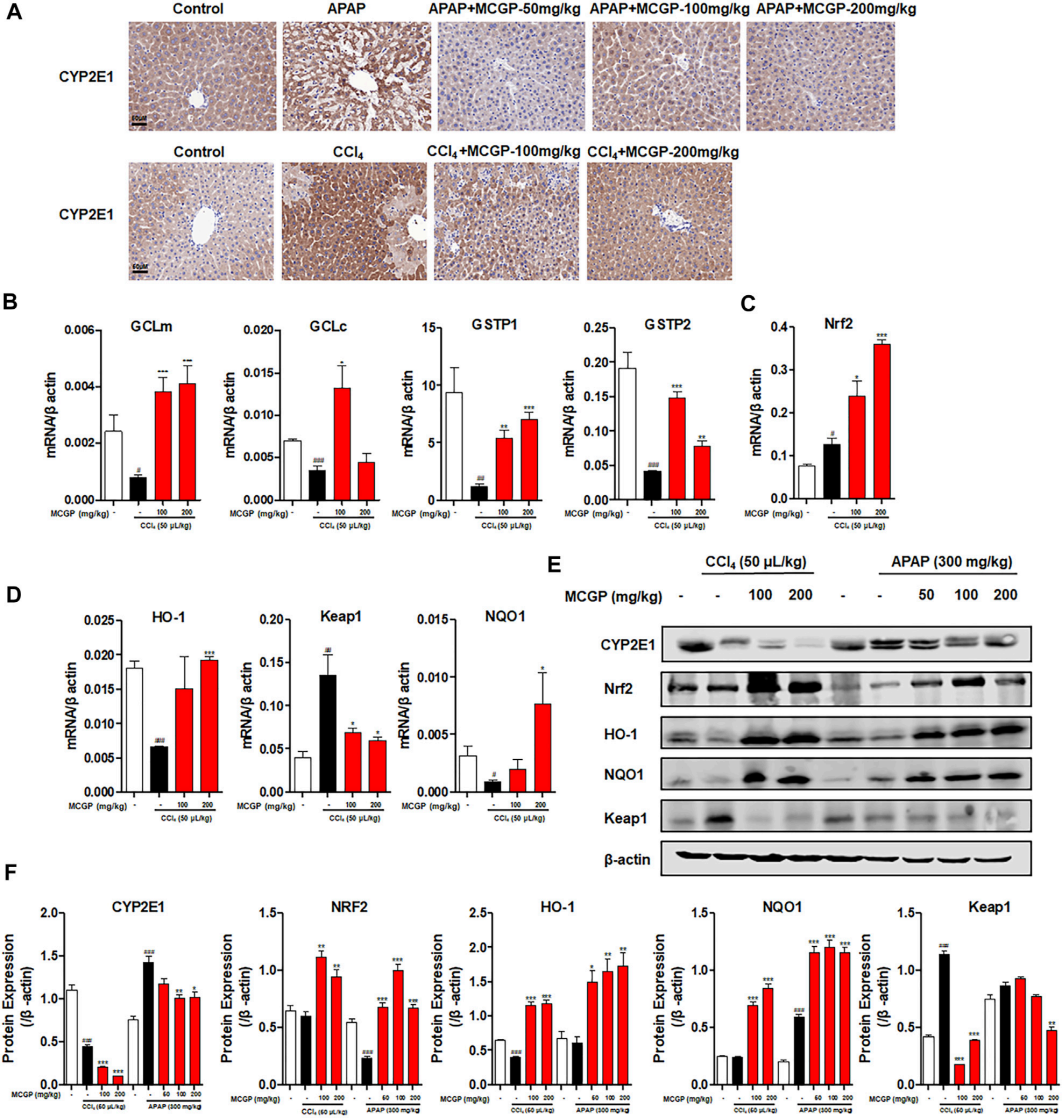
MCGP inhibited CYP2E1 and activated the NRF2/HO-1 signaling pathway in APAP-/CCl_4_-intoxicated mice. **(A)** Hepatic CYP2E1 immunohistochemical staining of each group in APAP- or CCl_4_-intoxicated mice. **(B-D)** Transcription level of GClm, GCLc, GSTP1, NRF2, HO-1, Keap1, and NQO1 of each group in CCl_4_-intoxicated mice. **(E,F)** The protein expression of CYP2E1, Nrf2, HO-1, NQO1, and Keap1 in liver tissues from the APAP or CCl_4_ groups **(E)**. The β-actin was used as an invariant control for equal loading, followed by the integrity optical density analyses of each blot **(F)**. Data were presented as the mean ± SEM (n = 3). #*p* < 0.05, ##*p* < 0.01, ###*p* < 0.001 vs control, *p < 0.05, **p < 0.01, ***p < 0.001 vs model.

A small amount of hepatotoxins (such as APAP and CCl_4_) are metabolized by cytochrome P450 enzymes, mainly *via* CYP2E1 isoform. APAP is metabolized by CYP2E1 to NAPQI, which exhausts GSH and leads to mitochondrial damages and necrotic cell death (Lancaster et al., 2015). Similarly, CCl_4_ is metabolized by CYP2E1 to highly reactive free radical metabolites that initiate membrane lipid peroxidation (Fahmy et al., 2016). The overactivation of CYP2E1 induced by APAP or CCl_4_ leads to oxidative stress and resultant liver damage. In our result, MCGP significantly depressed the protein increase of liver CYP2E1 on APAP-/CCl_4_-induced mice (Figures 6A,E). Glutamate–cysteine ligase catalytic subunit (Gclc) and glutamate–cysteine ligase modifier subunit (Gclm) are two subunits of glutamate–cysteine ligase, the first rate-limiting enzyme of glutathione synthesis (Jin et al., 2019). The glutathione S-transferase (GST) family includes glutathione S-transferase pi 1 (Gstp1) and glutathione S-transferase pi 2 (Gstp2), which play an important role in detoxification by catalyzing the conjugation of many hydrophobic and electrophilic compounds with reduced glutathione (Mari et al., 2009). CCl_4_ administration inhibits the transcription level of Gclc, Gclm, Gstp1, and Gstp2, while MCGP rescued their decrease (Figure 6B).

Oxidative stress is closely relevant with APAP-/CCl_4_-induced acute liver injury (Lu et al., 2016; Wang S. et al., 2017). Nrf2 is a transcription factor that plays an important role against oxidative stress by mediating the expression of many endogenous antioxidants such as GSTs, GCLs, and quinine oxidoreductase 1 (NQO1) (Bataille and Manautou 2012). MCGP significantly activated the gene and protein expression of Nrf2, NQO1, and HO-1 in APAP-/CCl_4_-induced mice liver (Figures 6C–F). In addition, MCGP suppresses the expression of kelch-like ECH associated protein 1 (Keap1) (Figures 6D–F), thus dissociating Nrf2 from Keap1 and leading to the nuclear translocation of Nrf2. These results are also consistent with the RNA-seq result that MCGP upregulated the transcription of Nrf2 signaling molecules (Figure 3F).

### Methyl 6-O-Cinnamoyl-α-d-Glucopyranoside Treatment Promoted Liver Regeneration and Protected Against Hepatocyte Apoptosis in Acetaminophen-/Carbon Tetrachloride-Intoxicated Mice

**FIGURE 7:**
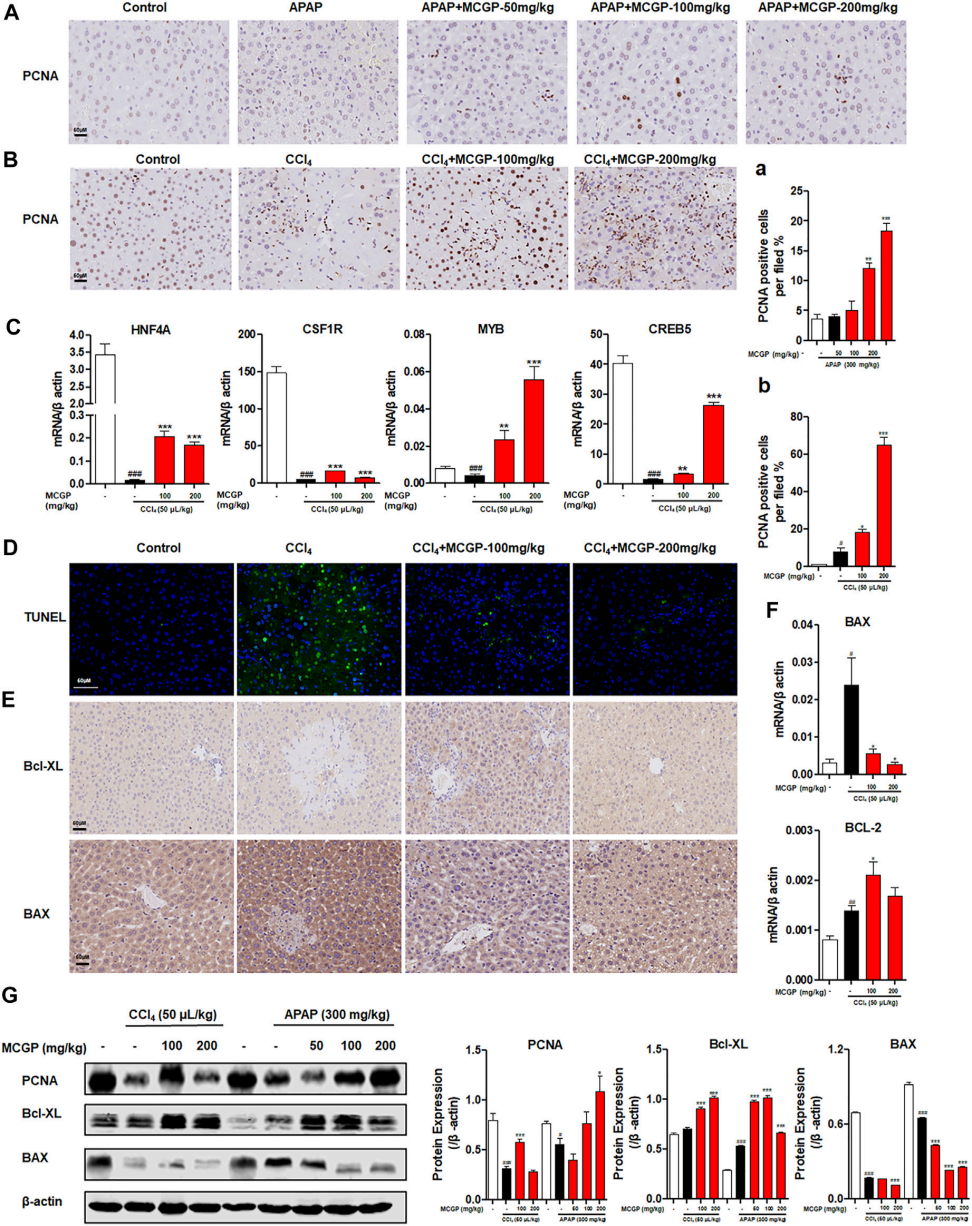
MCGP promoted liver regeneration and protected against hepatocyte apoptosis in APAP-/CCl_4_-intoxicated mice. **(A)** Hepatic PCNA immunohistochemical staining of each group in APAP-intoxicated mice. **(A)** The number of PCNA-positive hepatocytes per field. **(B)** Hepatic PCNA immunohistochemical staining of CCl_4_-intoxicated mice. **(B)** The number of PCNA-positive hepatocytes per field, scale bar: 50 μM. **(C)** Transcription level of HNF4A, CSF1R, MYB, and CREB5 of each group in CCl_4_-intoxicated mice. **(D)** TUNEL-staining of each group in CCl_4_-intoxicated mice, 200x. **(E)** Hepatic Bcl-XL and BAX immunohistochemical staining of each group in CCl_4_-intoxicated mice. **(F)** Transcription level of BAX and BCL-2 of each group in CCl_4_-intoxicated mice. **(G)** The protein expression of PCNA, Bcl-XL, and BAX in liver tissue from the APAP or CCl_4_ groups. The β-actin was used as an invariant control for equal loading, followed by the integrity optical density analyses of each blot. Data were presented as the mean ± SEM (n = 3). #*p* < 0.05, ##*p* < 0.01, ###*p* < 0.001 vs control, *p < 0.05, **p < 0.01, ***p < 0.001 vs model.

Nrf2 has been confirmed with the function on promotion of liver regeneration and inhibition of hepatocyte apoptosis (Chan et al., 2021; Ghanim and Qinna 2022). Therefore, we explored the effect of MCGP on liver regeneration and apoptosis. As shown in Figure 7A, both APAP and CCl_4_ increased the number of PCNA-staining hepatocytes in livers from APAP-/CCl_4_-intoxicated mice. Similarly, MCGP markedly increased the number of PCNA-staining hepatocytes in livers from APAP-/CCl_4_-intoxicated mice (Figures 7A,B). In addition, MCGP promoted the protein expression of PCNA in APAP-/CCl_4_-intoxicated mice livers (Figure 7G). Hepatocyte nuclear factor 4 alpha (HNF4A), colony stimulating factor 1 receptor (CSF1), MYB proto-oncogene, transcription factor (MYB), and cAMP responsive element binding protein 5 (CREB5) are downregulated genes revealed by RNA-seq after MCGP administration, while research studies showed that the four genes were related to liver regeneration, anti-apoptosis, and cell proliferation (Lancaster et al., 2015; Wang Y. et al., 2017; Wu et al., 2018; Zhou et al., 2020). As expected, we found the increase of these genes in MCGP-treated livers from CCl_4_-intoxicated mice, which is incompatible with the RNA-seq result (Figure 7C).

CCl_4_ exposure significantly increased hepatocyte apoptosis, while MCGP remarkably decreased the TUNEL-positive hepatocytes in livers from CCl_4_-intoxicated mice (Figure 7D). In addition, the apoptosis-related gene and protein expressions of Bax and Bcl-xL in the liver tissue were also detected by immunohistochemistry, qRT-PCR, and Western blotting. As a result, CCl_4_ had a slight effect on the expression of BCL2-like 1 (Bcl-XL) (Figures 7E,G), and significantly increased the transcription level of BCL2 apoptosis regulator (BCL-2) (Figure 7F). MCGP notably promoted the expression of Bcl-XL and BCL-2 in APAP-/CCl_4_-induced mice livers (Figures 7E–G). In addition, MCGP also decreased the gene and protein level of BCL2 associated X, apoptosis regulator (BAX) (Figures 7E–G).

### Methyl 6-O-Cinnamoyl-α-d-Glucopyranoside Treatment Promoted IL18 Secretion and Inhibited Inflammation in Acetaminophen-/Carbon Tetrachloride-Intoxicated Mice

**FIGURE 8:**
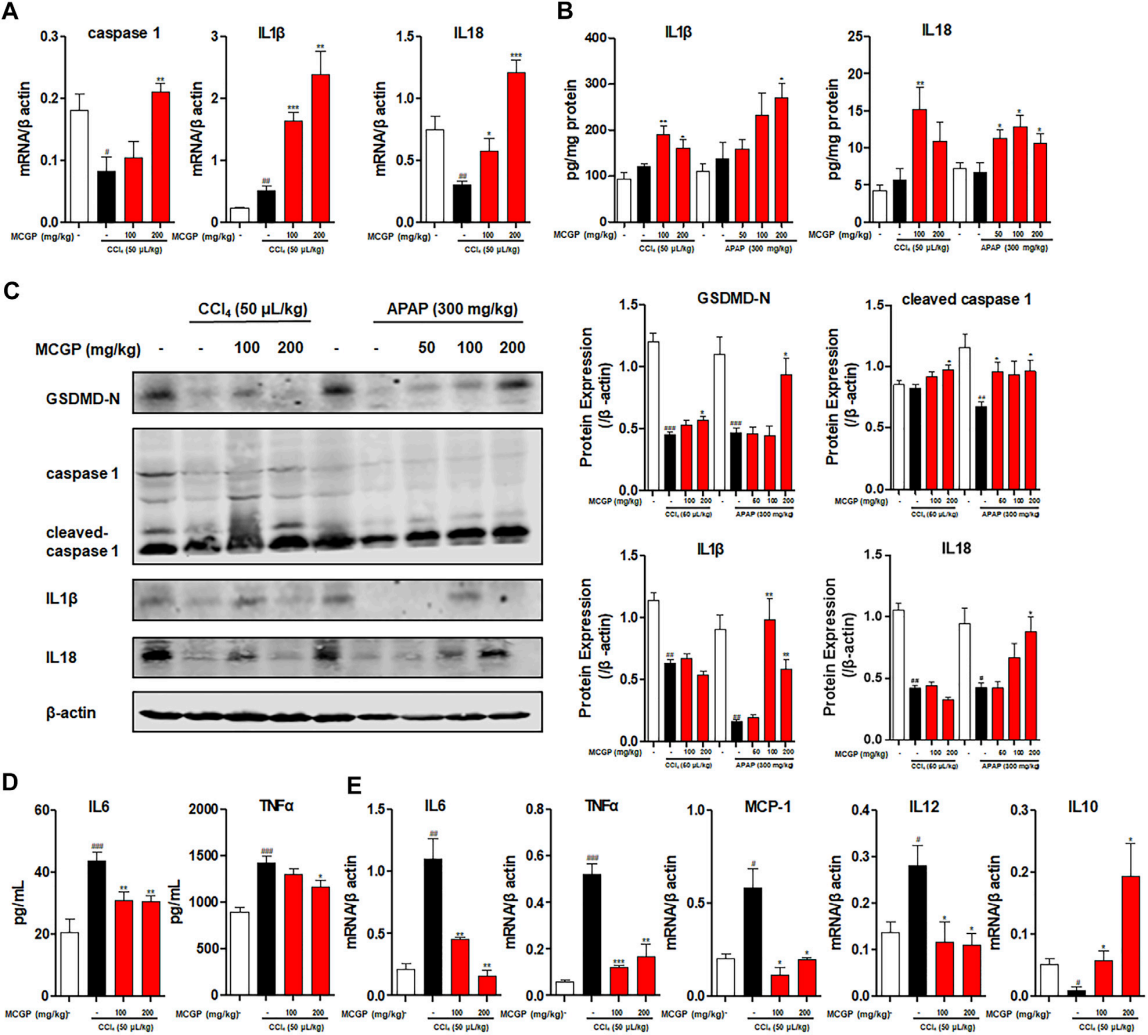
MCGP promoted IL18 secretion and inhibited inflammation in APAP-/CCl_4_-intoxicated mice livers. **(A)** Transcription level of caspase 1, IL1β, and IL18 of each group in CCl_4_-intoxicated mice. **(B)** The liver content of IL1β and IL18 of each group in APAP- or CCl_4_-intoxicated mice. **(C)** The protein expression of GSDMD-N, caspase 1, cleaved-caspase 1, IL1β, and IL18 in liver tissue from the APAP or CCl_4_ groups. The β-actin was used as an invariant control for equal loading, followed by the integrity optical density analyses of each blot. **(D)** The serum level of IL6 and TNFα of each group in CCl_4_-intoxicated mice. **(E)** Transcription level of IL6, TNFα, MCP-1, IL12, and IL10 of each group in CCl_4_-intoxicated mice. Data were presented as the mean ± SEM (n = 3). #*p* < 0.05, ##*p* < 0.01, ###*p* < 0.001 vs control, *p < 0.05, **p < 0.01, ***p < 0.001 vs model.

Previous studies revealed that IL18 accelerated liver regeneration after partial hepatectomy or APAP-induced liver injury (Zhang et al., 2014; Shi et al., 2020). IL-1β and IL-18 are two main pro-inflammatory cytokines produced during the recruitment of caspase 1 to cleaved GSDMD into its active form GSDMD-N and insert in cell membranes (de Vasconcelos et al., 2016; Shojaie et al., 2020). Here, we found that MCGP activated the transcription of caspase 1, IL1β, and IL18 and elevated the liver content of IL1β and IL18, inducing the protein expression of GSDMD-N, cleaved caspase 1, IL1β, and IL18 (Figures 8A–C).

APAP- or CCl_4_-induced liver injury is characterized by sterile inflammation, functioning as damage-associated molecular patterns (DAMPs), which can transcriptionally activate inflammatory cytokines (TNFα, IL1β, IL6, and IL10) and chemokines (MCP-1, MIP-2, and IL8) (Dambach et al., 2002; James et al., 2005). As a result, CCl_4_ markedly elevated the serum level of pro-inflammatory cytokines IL6 and TNFα (Figure 8D). In contrast, MCGP decreased the level of IL6 and TNFα (Figure 8A). In addition, MCGP inhibited the elevated transcription level of inflammation factors IL6, TNFα, MCP-1, and IL12 by CCl_4_, while it increased the expression of anti-inflammatory factor IL10 (Figure 8E).

## Discussion

In this study, we first identified the hepatoprotective effect of MCGP on two different types of acute liver injury animal models. Our results indicated that MCGP pretreatment efficiently alleviated intrahepatic hemorrhage, nuclear pyknosis, liver necrosis, and transaminase levels in APAP-/CCl_4_-intoxicated mice. Further exploration revealed that the effects of MCGP were relevant to inhibition of oxidative stress, hepatocyte apoptosis, and promotion of liver regeneration.

APAP- or CCl_4_-induced acute liver injuries are widely used to explore novel liver-protective agents. CYP2E1 catalyzes APAP to its toxic intermediate NAPQI, and excess NAPQI causes significant depletion of GSH (Lancaster et al., 2015). GSH can protect cells from oxidative damage by scavenging free radicals and other oxygen species through nonenzymatic and enzymatic processes (Lancaster et al., 2015). Overloaded NAPQI covalently conjugated to sulfhydryl groups in cellular proteins triggers mitochondrial oxidative stress and dysfunction, which ultimately initiates hepatocellular apoptosis and necrosis (Lancaster et al., 2015). CCl_4_ is also metabolized into radicals CCl_3_• and OOCCl_3_• by CYP2E1.^32^ Abundant radicals induce lipid peroxidation and ROS generation, ultimately causing hepatocyte necrosis and liver dysfunction (Fahmy et al., 2016). In this study, we demonstrated that MCGP can increase the liver level of SOD and GSH, inhibit the expression of CYP2E1, and promote the transcription level of glutathione synthesis rate-limiting enzymes (GCLs) and glutathione transferases (GSTPs), thus protecting liver from oxidative damage. In addition, MCGP also decreased the ROS content in H_2_O_2_-stimulated murine normal liver cell AML12. However, MCGP could not directly inhibit ABTS^•+^ radical by *in vitro* oxidation–reduction reactions, which suggested that MCGP might suppress oxidative stress through cellular progress. Nrf2 signaling pathway is a classical way for antioxidants. Activation of Nrf2 transcribes antioxidant enzymes, including microsomal epoxide hydrolase, HO-1, NQO-1, and glutamate GCL, acting as a cell defense system to detoxify NAPQI (Zhao et al., 2021). Further results exhibited that MCGP promoted the protein expression of Nrf2, HO-1, and NQO1 and inhibited the expression of Keap1. Taking together, we presume that MCGP can ameliorate APAP/CCl_4_-induced oxidative stress through the inhibition of CYP2E1 and the activation of the Nrf2 signaling pathway. These results were consistent with the improvement of liver antioxidant enzymes SOD, GSH, and MDA.

Hepatocyte apoptosis is a representative attribution of acute liver injury induced by oxidative stress; APAP or CCl_4_ is reported to induce notable elevation of hepatocyte apoptosis (Lu et al., 2016; Yan et al., 2016). In this study, we exhibited that APAP or CCl_4_ obviously increased the number of TUNEL-positive cells, while MCGP significantly decreased apoptotic hepatocytes. Consistent with the decreased TUNEL-positive cells, apoptosis-related protein Bax was down-expressed while Bcl-XL was up-expressed on MCGP pretreated APAP-/CCl_4_-intoxicated mice livers. Apart from this, MCGP also ameliorated the cell viability of APAP- or CCl_4_-intoxicated AML12 cells and decreased the number of apoptotic cells. All these results suggested that MCGP significantly ameliorated APAP- or CCl_4_-induced liver damage by restraining hepatocyte apoptosis induced by oxidative stress.

On the other hand, the restoration of liver normal function after acute liver injury can be acquired through promoting liver regeneration, which is proved to be efficient in APAP- or CCl_4_-induced liver injury (Yan et al., 2018; Humpton et al., 2021). In addition, activation of Nrf2 enhances functional liver regeneration (Chan et al., 2021; Ghanim and Qinna 2022). Hence, we supposed that MCGP could also promote liver regeneration through the activation of the Nrf2 signaling pathway. PCNA and HNF4A were reported to be critical on regulating liver regeneration (Sherr 1994; Lee et al., 2020). CSF1R and its ligand colony stimulating factor 1 (CSF1) regulate the proliferation, differentiation, and function of macrophages, including Kupffer cells (Santamaria-Barria et al., 2019). In damaged tissues, CSF1R and CSF1 assist macrophages to suppress immune response and promote vascular regeneration to accelerate repairing; inhibition of CSF1R delays liver regeneration (Santamaria-Barria et al., 2019). MYB, CREB5, and BCL-2 are molecules in the PI3K/AKT signaling pathway, which is relevant to cell survival and proliferation (Lin et al., 2020). Our results showed that MCGP pretreatment can markedly promote the PCNA-positive cells and increase the expression of PCNA, HNF4A, CSF1R, MYB, and CREB5 in livers. These results indicate that MCGP can, on the one hand, inhibit APAP-/CCl_4_-induced hepatocyte apoptosis by regulating the liver expression of apoptosis-related proteins, and on the other hand, promote liver repairing and regeneration by motivating the expression of cell survival and proliferation protein.

Reports revealed that necrosis of hepatocytes trigger the releasing of inflammatory mediators such as cellular contents, which can function as DAMPs (Ramachandran and Jaeschke 2019). DAMPs can activate Kupffer cells to generate large amounts of cytokines, thus recruiting circulating neutrophils and monocytes into the liver, resulting in the initiation of inflammatory-related injury (Ramachandran and Jaeschke 2019). The major function of NOD-like receptor pyrin domain containing 3 (NLRP3) inflammasome is to recognize a wide variety of danger signals including DAMPs, and thus lead to the activation of caspase-1, which further conducts the production of mature IL-1β and IL-18 (Yang et al., 2019). A previous study also covered the crucial role of Nrf2 on the protection effect of baicalin on APAP-induced liver injury (Shi et al., 2018). The follow-up report from the group reported that baicalin promoted the interaction between Nrf2 and Nlrp3, ASC, and caspase 1 in APAP-intoxicated mice, thus leading to the assembly of NLRP3 inflammasome and the subsequent IL-18 product, thereby promoting hepatocyte proliferation after APAP-induced acute liver injury (Shi et al., 2020). In our study, MCGP administration promoted the activation of cleaved caspase 1, GSDMD-N, IL1β, and IL18.

IL-1β and IL-18 are two typical pro-inflammatory cytokines produced during NLRP3 inflammasome activation. However, the promotion of IL1β and IL18 by MCGP did not enhance liver inflammation but reduced liver inflammation in CCl_4_-intoxicated mice. In addition, administration of high pharmacological doses of IL1β directly do not aggravate APAP-induced liver injury (Williams et al., 2010). Moreover, previous studies revealed that IL18 accelerated liver regeneration after partial hepatectomy or APAP-induced liver injury (Zhang et al., 2014; Shi et al., 2020). In this study, MCGP can significantly increase the liver IL1β and IL18 protein expression. In addition, MCGP enhanced the amount of hepatic IL1β and IL-18 in APAP-/CCl_4_-intoxicated mice. Taking together, we suspect that MCGP ameliorated acute liver injury partially through the promotion of IL18 content to accelerated liver regeneration.

In conclusion, this study, for the first time, identified the protective effect of MCGP in APAP-/CCl_4_-intoxied acute liver injury. Mechanically, MCGP may exert its hepatoprotective effect through the alleviation of oxidative stress, the suppression of hepatocyte apoptosis, and the promotion of liver regeneration. Furthermore, these results also suggest that MCGP may be protective against other types of acute liver injury, such as those induced by lipopolysaccharide or concanavalin A, which is what we are identifying at present.

## Data Availability

The raw sequences of this study have been deposited in the Sequence Read Archive (accession number: SRR18347304 and SRR18347305).

## Abbreviations

MCGP, methyl 6-O-cinnamoyl-α-d-glucopyranoside; APAP, acetaminophen; CCl_4_, carbon tetrachloride; PCNA, proliferating cell nuclear antigen; CYP2E1, cytochrome P450 family 2 subfamily E member 1; ROS, reactive oxygen species; NAPQI, N-acetyl-p-benzoquinone imine; GSH, glutathione; CCl_3_−, trichloromethyl; Nrf2, nuclear factor erythroid 2-related factor 2; NQO1, NAD(P)H quinine oxidoreductase 1; SOD, superoxide dismutase; HO-1, heme oxygenase 1; GST, glutathione S-transferase; HNF4A, hepatocyte nuclear factor 4 alpha; CSF1, colony stimulating factor 1 receptor; MYB, MYB proto-oncogene, transcription factor; GSTs, glutathione S-transferases; Gclc, glutamate-cysteine ligase catalytic subunit.
