# Supplementary material for: Methyl 6-O-cinnamoyl-α-d-glucopyranoside Ameliorates Acute Liver Injury by Inhibiting Oxidative Stress Through the Activation of Nrf2 Signaling Pathway
Source: Front Pharmacol. 2022 Apr 26;13:873938. doi: 10.3389/fphar.2022.873938 (PMC9086595; doi:10.3389/fphar.2022.873938)
Supplement: Supplementary file 1 [file Table1.DOCX]

**Supporting Information**

**Methyl 6-O-cinnamoyl-α-D-glucopyranoside (MCGP) ameliorates acute liver injury by inhibiting oxidative stress through the activation of NRF2 signaling pathway**

Qianqian Xu^1, #^, Yanfang Deng^1, #^, Jiaxiong Ming^4^, Zengwei Luo^1^, Xia Chen^3^, Tianqi Chen^1^, Yafen Wang^1^, Shan Yan^1^, Jiajun Zhou^1^, Lina Mao^1^, Weiguang Sun^1, *^, Qun Zhou^1, *^, Hong Ren^2, *^, Yonghui Zhang^1, *^

^1^ Hubei Key Laboratory of Natural Medicinal Chemistry and Resource Evaluation, School of Pharmacy, Tongji Medical College, Huazhong University of Science and Technology, Wuhan 430030, China;

^2^ Biobank, Union Hospital, Tongji Medical College, Huazhong University of Science and Technology, Wuhan 430022, China;

^3^ National & Local Joint Engineering Research Center of High-throughput Drug Screening Technology, Hubei Key Laboratory of Biotechnology of Chinese Traditional Medicine, School of Life Sciences, Hubei University, Wuhan, 430062, China.

^4^ Department of Pharmacy, School of Medicine, Wuhan College of Arts & Sciences, Wuhan 430345, China

^*^Corresponding authors at: Hubei Key Laboratory of Natural Medicinal Chemistry and Resource Evaluation, School of Pharmacy, Tongji Medical College, Huazhong University of Science and Technology, Wuhan 430030, China; Biobank, Union Hospital, Tongji Medical College, Huazhong University of Science and Technology, Wuhan 430022, China.

E-mail addresses: zhangyh@mails.tjmu.edu.cn (Y. Zhang), renhong@hust.edu.cn (H. Ren), zqtcm@163.com (Q. Zhou), [weiguang_s@hust.edu.cn](mailto:weiguang_s@hust.edu.cn) (W. Sun).

^#^These authors contributed equally to this work.

**Supplementary Table 1. List of human and mouse gene primers for qRT-PCR.**

| Mouse Gene | Sense | Antisense |
| --- | --- | --- |
| β-actin | CGTGCGTGACATCAAAGAGAA | TGGATGCCACAGGATTCCAT |
| HNF4A | CAAGAAGCCATCTAGCAAGCTG | TTGCCATGACTAGGTTACTCCC |
| CSF1R | GGTTCATTATCCGCAAGGCTA | CCTTAAGCCAGATGCCAGT |
| MYB | CCCTATCCTGTCGCATTGCAT | TCCTTCTCAGGGTCTTCGTCGTT |
| CREB5 | AGATCAGACTCCGACGCCCAC | AGCCTTCCTGAACTCGTGCTC |
| BAX | TGGTTGCCCTCTTCTACTTTGC | TGTCCAGCCCATGATGGTTC |
| BCL-2 | CCCCTTCATCCAAGAATGCAA | TCTCCCGGTTATCATACCCTG |
| GCLm | CAATGACCCGAAAGAACTGCT | AGCCTTTAGACTTGATGATTCCC |
| GCLc | AACTCTGCCTATGTGGTATTCGT | GGGCCACTTTCATGTTCTCGT |
| GSTP1 | TTCGCGGCAAATATGTCACC | CCTTCACGTAGTCATTCTTACCA |
| GSTP2 | CTAATGCCATCTTGAGACACC | TATTTGCCGCGAAGGTCCTC |
| Nrf2 | GCTCCTATGCGTGAATCCCA | TTTTGCCCTAAGCTCATCTCGT |
| HO-1 | AGGTACACATCCAAGCCGAGA | AGCCATCACCAGCTTAAAGCC |
| Keap1 | TAACCGGCTTAACTCCGCAGA | GTGTGATCATCCGCCACTCA |
| NQO1 | GACAACGGTCCTTTCCAGAATA | CTCTGAATCGGCCAGAGAATG |
| caspase 1 | AACAACCAGAATTTAGGCTAC | GCTTCTTATTGGCACGATT |
| IL1β | CTACCTGTGTCTTTCCCGTG | CCTGTAGTGCAGTTGTCTAAT |
| IL18 | ACACGCTTTACTTTATACCTG | AACTTGGTCATTTATATTCCGTA |
| IL6 | TAAAATAGTCCTTCCTACCCC | TTGCCGAGTAGATCTCAAA |
| TNFα | CGTCGTAGCAAACCACCAAGT | CCATCGGCTGGCACCACTA |
| MCP-1 | CTACTCATTCACCAGCAAGAT | CCATTCCTTCTTGGGGTCA |
| IL12 | TGGCTACTAGAGAGACTTCTT | ATAGATGCTACCAAGGCACAG |
| IL10 | CAACATACTGCTAACCGACT | CTGGATCATTTCCGATAAGGC |

**General Procedure for Synthesis of MCGP**

To a stirred solution of the cinnamic acid (17.83 g, 120 mmol, 1.0 equiv.) in anhydrous CH_2_Cl_2_ (50 mL) under inert atmosphere was added dropwise oxalyl chloride (20 mL, 240 mmol, 2.0 equiv.) and followed by a catalytic amount of anhydrous DMF (0.19 mL, 2.4 mmol, 0.02 equiv.). Then the reaction was allowed to warm to room temperature and stirred for about 6 hr and monitored by TLC analysis. The solvent was then removed under reduce pressure to afford the corresponding cinnamoyl chloride (yellow oil), which was used directly for the next step without further purification.

The in situ generated cinnamoyl chloride (18.8 g, 113 mmol, 1.1 equiv.) was dissolved in anhydrous CH_2_Cl_2_ (50 mL) and was added dropwise to a mixture of methyl-α-D-glucopyranoside (20.0 g, 103 mmol, 1.0 equiv.) and anhydrous pyridine (50 mL) at -10 °C and stirred for 30 min. Then the reaction was allowed to warm to room temperature and stirred for about 6 h and monitored by TLC analysis. The solvent was removed under reduce pressure to afford the corresponding reaction mixture, then it was further concentrated by a vacuum pump for 1 h to remove the residual solvent. The reaction mixture was quenched by water and extracted with ethyl acetate for three times. The combined organic layer was dried over anhydrous Na_2_SO_4_, filtered and concentrated. The residue was purified by column chromatography on silica gel to afford MCGP (white solid, 12.69 g, yield: 38%).

**Characterization of MCGP**

Methyl 6-O-cinnamoyl-α-D-glucopyranoside (MCGP)

MCGP (White solid): 1H NMR (CD3OD, 400 MHz, Refer to H 3.31) H 3.36 (dd, J = 9.7, 9.2 Hz, 1H, H-4), 3.42 (s, 3H, 1-OCH3), 3.44 (dd, J = 9.7, 3.9 Hz, 1H, H-2), 3.65 (t, J = 9.0 Hz, 1H, H-3), 3.79 (ddd, J = 10.0, 5.9, 2.1 Hz, 1H, H-5), 4.34 (dd, J = 11.9, 6.0 Hz, 1H, H-6a), 4.51 (dd, J = 11.9, 2.2 Hz, 1H, H-6b), 4.68 (d, J = 3.7 Hz, 1H, H-1), 6.55 (d, J = 16.0 Hz, 1H, H-2’), 7.39-7.41 (m, 3H, H-6’, H-7’, H-8’), 7.59-7.61 (m, 2H, H-5’, H-9’), 7.71 (d, J = 16.0 Hz, 1H, H-3’); 13C NMR (CD3OD, 100 MHz, Refer to C 49.0) C 55.6 (1-OCH3), 64.9 (C-6), 71.1 (C-5), 71.9 (C-4), 73.5 (C-2), 75.0 (C-3), 101.3 (C-1), 118.6 (C-2’), 129.3 (2xC-Ar), 130.0 (2xC-Ar), 131.6 (C-Ar), 135.7 (C-4’), 146.5 (C-3’), 168.5 (C-1’).

**^1^H NMR spectrum of MCGP**

**^13^C NMR spectrum of MCGP**

**DEPT 135 spectrum of MCGP**
